# A protocol of histone modification-based mechanistic study of acupuncture in patients with stable angina pectoris

**DOI:** 10.1186/s12906-015-0653-0

**Published:** 2015-04-30

**Authors:** Ning Wang, Sheng-Feng Lu, Hui Chen, Jian-Fei Wang, Shu-Ping Fu, Chen-Jun Hu, Yi Yang, Fan-Rong Liang, Bing-Mei Zhu

**Affiliations:** Key Laboratory of Acupuncture and Medicine Research of Ministry of Education, Nanjing University of Chinese Medicine, 210023 Nanjing, China; School of Acupuncture and Tuina, Chengdu University of Traditional Chinese Medicine, 610075 Chengdu, Sichuan China

**Keywords:** Acupuncture, Stable angina pectoris, Study protocol, Gene profiling, Histone modification

## Abstract

**Background:**

Angina pectoris (Angina) is a medical condition related to myocardial ischemia. Although acupuncture has been widely accepted as a clinical approach for angina, there is no sufficient evidence of its effectiveness against this syndrome, and its mechanisms have not yet been well elucidated. We develop this protocol to confirm the clinical efficacy of electro-acupuncture on stable angina pectoris by needling on acupoint Neiguan (PC6). Furthermore, we employ high-throughput sequencing technology to investigate the gene expression profiling and determine involvement of histone modifications in the regulation of genes after electro-acupuncture treatment.

**Methods/Design:**

A randomized, controlled, double-blinded (assessor and patients) trial will be carried out. Sixty participants will be randomly assigned to two acupuncture treatment groups and one control group in a 1:1:1 ratio. Participants in acupuncture groups will receive 12 sessions of electro-acupuncture treatment across 4 weeks, followed by a 12-week randomization period. The acupuncture groups are divided into Neiguan (PC6) on Pericardium Meridian of Hand-jueyin or a non-acupoint. The primary clinical measure of effect is the frequency of angina attacks between these groups for four weeks after randomization. RNAs are extracted from peripheral neutrophils collected from all participants on day 0, day 30, and week 16, and are processed to RNA-Seq. We then investigate profiles of histone modifications by ChIP-Seq, for H3 Lysine 4 (H3K4me) and acetylation of H3 Lysine 27 (H3K27ac), in the presence or absence of acupuncture treatment.

**Discussion:**

This study determines the efficacy and mechanisms of electro-acupuncture on stable angina pectoris. We focus on effectiveness of acupuncture on alleviating symptoms of myocardial ischemia and the gene regulation and the chromatin remodeling marks, including H3K4me1, H3K4me2, and H3K27ac, which could be key factors for regulating gene expressions caused by electro-acupuncture treatment at Neiguan. This is the first genome-wide study of electro-acupuncture treatment in angina patients, and will provide valuable information for future studies in the fields of acupuncture and its underlying mechanisms.

Fourteen patients have been recruited since recruitment opened in November of 2012. This study is scheduled to end in November of 2014.

**Trials registration:**

ChiCTR-TRC-12002668

## Background

Stable angina is a clinical syndrome characterized by discomfort in the chest, jaw, shoulder, back, or arms, typically elicited by physical exertion or emotional stress and relieved by rest or nitroglycerin [1]. Despite the multiple medical and interventional technologies that have been developed to reduce myocardial ischemia, stable angina still affects nearly 60 million people in China. And despite the association with a worsened quality of life [2], repeated hospitalizations, and increased healthcare costs [3], angina often remains undertreated [4,5]. Acupuncture, the most well-known complementary and alternative medical approach, has been applied to prevent and cure many diseases such as angina [6], palpitation [7], stroke [8], and disruption of the left cardiac function in coronary heart disease (CHD) [9] for more than 2000 years. Recent studies show that acupuncture treatment can improve regeneration capacity of local micro-vascular and collateral circulation during myocardia ischemia; thereby ameliorating ischemia symptoms [10-13]. It not only quickly relieves the symptoms of acute angina pectoris, but also improves nitroglycerine’s therapeutic effects [14]. A review article has concluded that the effectiveness (between 80% to 96.2%) of acupuncture therapy is comparable to conventional drug regimen [14]. In addition, many conventional anti-angina medications react adversely with other medications that the patients may be taking for other illnesses, whereas acupuncture therapy does not pose such an interference with the patient’s medical regimen. Clinical experience has suggested that myocardial ischemia can be effectively treated via acupuncture at a single acupoint, Neiguan (PC6), which is first described in Miraculous Pivot Meridians. Neiguan (PC6) indicates a point located 2 cun above the transverse crease of the wrist and between the tendons of m. Palmaris longus and m. flexor radialis. And because this point is connected to the pericardium, it can also be used to treat precordial pain [15]. Neiguan (PC6) is the Luo point of the pericardium meridian of hand-jueyin, as well as one of the eight confluence acupoints.

Many have proven that needling at PC6 can protect against myocardial ischemic injury through multiple mechanisms, including improving angiogenesis and reducing apoptosis and calcium overload [16-18]. Though some studies have looked at the systemic alternations of functional molecules after median nerve stimulation in rats with myocardial ischemic injury [19], no epigenetic research has been conducted on the response to electro-acupuncture (EA) treatment applied to angina patients. Epigenetic modifications of DNA and histones are a primary mechanism by which gene expression activities may be modified in response to environmental stimulation. Due to the innate plasticity of DNA methylation of cytosine bases, environmental cues can induce epigenetic shifts in the timing and intensity of gene expression that may contribute to a physiological acclimation response [20]. Although the vast majority of modifications remain poorly understood, scientists have seen considerable progress in the understanding of lysine acetylation and methylation. Whereas lysine acetylation almost always correlates with chromatin accessibility and transcriptional activity, lysine methylation can have different effects depending on which residue is modified. Methylation of histone H3 lysine 4 (H3K4) and H3 lysine 36 is associated with transcribed chromatin. In contrast, methylation of H3 lysine 9 (H3K9), H3 lysine 27 (H3K27), and H4 lysine 20 (H4K20) generally correlate with repression [21]. Recent studies show that the acetylation of lysine residues on histones is a well-established post-translational modification, and is a direct regulator of chromatin structure and function [22]. Two enzyme families, histone acetylase and histone de-acetylase, control the level of histone acetylation and act as critical gene transcriptional activators or silencers. It is known that post-translational modification of histone H3K9 is critical for regulation of gene transcription, and includes both acetylation and methylation. Methylation of histone H3K9 correlates with transcriptional repression, whereas acetylation of the histone H3K9 is associated with transcriptional activation [23]. Our previous studies on rats have demonstrated that acupuncture promotes angiogenesis after myocardial ischemia though regulation of H3K9 acetylation at the VEGF gene [17]. This is the first experimental report showing that acupuncture can effectively up-regulate VEGF expression through H3K9 acetylation modification directly at the VEGF promoter and hence contribute to angiogenesis in the rat MI model. A recent study demonstrates that enhancers play a central role in cell type-specific gene expression and are marked by H3K4me1/2. Active enhancers are further marked by H3K27ac. Using adipogenesis and myogenesis as model systems, Dr. Ge, et al. at NIH identified that deletion of Mll4 (KMT2D) markedly decreased H3K4me1/2 and H3K27ac levels on enhancers and leads to severe defects in cell type-specific gene expression and cell differentiation [24]. Moreover, others have found that histone modification-mediated chromatin remodeling plays an important role in the occurrence of cardiovascular disease [25]. We now aim to study whether H3K4me1/2 and H3K27ac can be correlated with the protective effects of acupuncture at PC6 in human patients.

A few published randomized controlled trials (RCTs) concerning acupuncture treatment for stable angina pectoris claim that convincing evidence for the effectiveness of acupuncture in treating stable angina pectoris patients is still inadequate, due to the poor quality of existing studies. To address these problems and to hopefully provide a more conclusive answer to these questions, a team within our research group has designed a protocol to start a multicenter RCT [26]. In order to study the underling molecular mechanisms, we will first confirm the efficacy of electro-acupuncture as a treatment method for stable angina pectoris using a relatively small patient population. We will then compare the gene profiles and histone modifications before and after electro-acupuncture treatment at Neiguan. This work is financially supported by the National Basic Research Program (973 program, No. 2012CB518501) of China.

## Methods/Design

### Clinical trial

**Figure 1:**
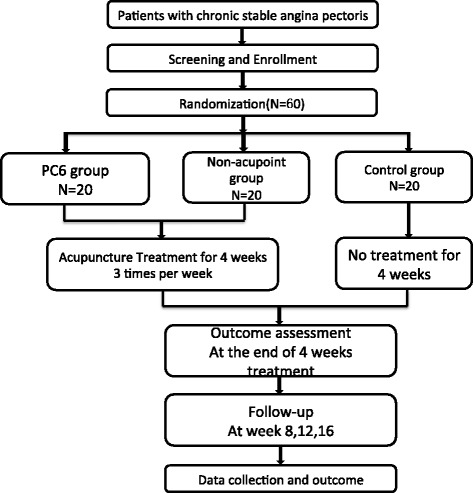
Trial flow chart.

This is a randomized controlled, double-blinded (assessor and patients) trial containing two acupuncture groups (Neiguan and Non-acupoint group) and one control group (Figure 1). Sixty participants will be included from the following two hospitals: Second Affiliated Hospital of Nanjing University of Chinese Medicine; and Affiliated Hospital of Hunan University of Chinese Medicine. These participants will be randomly assigned to three groups on a 1:1:1 ratio.

The two acupuncture groups will receive 12 sessions of electro-acupuncture treatment spanning 4 weeks, with the frequency of 3 sessions a week. Each session will last 30 minutes. The follow-up period is 3 months (5 to 16 weeks after randomization). The control group will not receive acupuncture treatment in the first 4 weeks but will receive routine clinical care in the hospital. They will be provided 12 sessions of electro-acupuncture treatment after the 16 weeks as compensation. Participants will be asked to fill in angina diaries from one week before randomization to 16 weeks after randomization. All clinical outcomes are assessed at baseline and at 4, 8, 12, and 16 weeks after randomization according to the diaries. The laboratory results are assessed at baseline, 4 weeks, and 16 weeks after randomization.

### Experimental study

**Figure 2:**
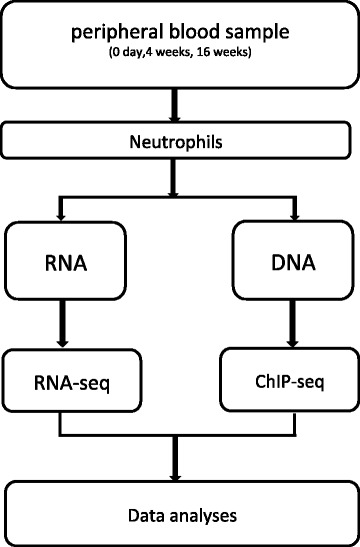
Experimental study design.

Ten ml peripheral blood will be drawn from each participant on the day 0, day 30, and at the end of week 16 (Figure 2). All neutrophils are negative selected from the peripheral blood. After extracting RNA and DNA from the blood sample, all of the data will be analyzed by high-throughput sequencer Illumina Hiseq 2000 (Illumina, USA) in our laboratory. We will analyze the RNA-Seq and ChIP-Seq data after 30 days of electro-acupuncture treatment. RNA-seq analysis from the experimental patients will provide acupuncture-related changes in gene expression and pathway profiles. Meanwhile, RNA-seq data generated from the control group will be used to eliminate background. To detect histone modifications, we perform ChIP-seq analysis for H3k4me1/2 and H3k27ac.

The total research period for this study is 16 weeks for each patient. All patients are asked to record angina diaries for one week before randomization (baseline phase). If they prove eligible for this study, they will be asked to complete diaries from 0 to 16 weeks after randomization. Each group will receive basic treatments including health education and basic drug therapy. We will recommend, to all patients, lifestyle modifications that include increasing physical activity, limiting alcohol consumption, controlling body weight, and quitting smoking, etc. The basic medications include Aspirin: 100 mg quaque die; Metoprolol: 25 mg bis in die; Ramipril: 5 mg quaque die; and Atorvastatin: 20 mg quaque nocte.

### Ethics review and informed consent

This protocol has been approved by the Chinese Clinical Trail Registry (ChiCTR) on Nov. 9,2012 (Registration number: ChiCTR-TRC-12002668). It follows the principles of the Declaration of Helsinki (Version Edinburgh 2000). It has been reviewed and approved by the Institutional Review Board of the Second affiliated Hospital Ethic Committee of Nanjing University of Chinese Medicine (Approved No.: 2012CB518501) and the First Affiliated Hospital Ethic Committee of Hunan University of Chinese Medicine (Approved No.: HN-LL-KY-2013-006-01). Before randomization, all patients will be requested to sign the written informed consent, during which they will be given enough time to decide whether they are willing to participate in this trial. They will be informed of the details of the study and all benefits and risks of participating in this trial.

## Study population and recruitment

Sample size is calculated based on the primary clinical measure of effect-frequency of angina attacks, according to the formula:

$$ \mathrm{n}=\frac{2{\left({u}_{\alpha }+{u}_{\beta}\right)}^2s{c}^2}{\delta^2} $$

Where n is the sample size of trial group/control group, α is the significance level,1-β is the power of a test, δ is the mean difference between trial group and control group. In this study, α = 0.05, β = 0.10, δ is estimated at 4.5 between Neiguan group and control group based on the primary clinical data, therefore n ≈ 20 is in each group. Thus, a total of 60 participants were recruited in outpatient clinics from the two above-mentioned hospitals. The chief physicians of cardiovascular department in each hospital were invited to attend a discussion on how to recruit patients. Research assistants from each department will help screen participants. To ensure the precision of results of this study, we developed the following criteria of eligibility.

### Inclusion criteria

Participants will be included in this study if they fulfill all of the following conditions: 1) Patient was diagnosed with stable angina according to standards published by the American College of Cardiology (ACC) and the American Heart Association (AHA) [27]. 2) Patients ages between 35 and 80 years old, both male and female; 3) The patient has experienced angina attacks in at least the last three months, and the attack frequency was equal to or greater than twice a week in the most recent month; 4) Patient signs the informed consent document. In addition, to ensure that the results from this study are only due to the treatments from this study, all participants will be required not to have received acupuncture therapy for at least 2 months prior to entering the study.

### Exclusion criteria

Patients with any of the following conditions will be excluded: 1) Pregnant or nursing mothers, including those who have given birth in the last six months; 2) Patients with pre-existing severe cardiovascular, digestive, urinary, respiratory, hematological, nervous, or endocrine system diseases that cannot be controlled with clinical treatments; 3) Patients with psychosis; 4) Patients who are susceptible to bleeding disorders and/or allergic reactions; 5) Patients with acute coronary syndrome (including acute myocardial infarction, unstable angina), severe arrhythmia (including 3rd degree AV block, ventricular tachycardia, sever supraventricular tachycardia, and frequent premature contractions, especially premature ventricular contraction), atrial fibrillation, primary myocardiopathy, or valvular heart disease; 6) Patients who have experienced unsuccessful treatments for hypertension or diabetes mellitus (average blood pressure above 140/90 mmHg and HbA1 above 7% in last three months); or 7) Patients participating in other clinical projects.

### Randomization and blinding

Patients will be randomized by an independent statistician using a computerized, random-number generator through the block-randomization method of Statistics Analysis System version 9.2 for sequence generation. Allocation to the treatment groups uses a stratified block dynamic randomization method with a permuted block, which is a computer-based system [26]. Separate randomization files will be created for each of the two recruiting sites. Allocations will be concealed using sealed, opaque envelopes. After the assessor has screened the patient for inclusion and the patient has signed the informed consent form, the next envelope in the sequence will be provided, immediately prior to treatment, to the certified acupuncturists, who will be conducting acupuncture treatment. With this method, the 60 participants will be randomly assigned to the three groups: Neiguan (PC6) group, non-acupoint group, and control group. The acupuncturists and the patients are not involved in the assessments of study results. Blinded assessors are required to conduct outcome assessments, collect outcome data and angina diaries, and check any missing data. Laboratory analysts are also blinded to the clinical evaluation and index. The data are entered by research assistants and analyzed by a statistician. Except for the acupuncturists, all personnel will not be disclosed information regarding participant allocation. Professional statistical assistance is also sought to double-check the data analysis.

### Interventions and comparison

#### Rationale for acupuncture protocol

**Figure 3:**
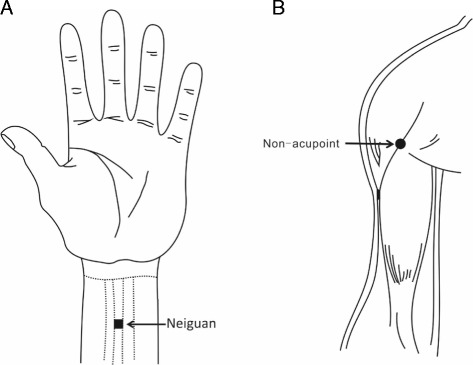
Illustration of acupoints. Location of the acupoint Neiguan (Figure 3A). Location of non-acupoin (Figure 3B).

The acupuncture protocol was set up upon consulting acupuncture experts and a variety of acupuncture textbooks. Based on the theory of acupuncture and Traditional Chinese Medicine (TCM), Neiguan (PC6) (Figure 3A) on Pericardium Meridian of Hand-jueyin is the classic acupoint in the treatment of stable angina pectoris. Additionally, to test and verify the clinical efficiency of Neiguan, we have chosen a non-acupoint (Figure 3B) located on the junction of the deltoid and biceps muscles at the front of medial upper arm that does not belong to any channel as a reference point. Therefore, acupuncture groups in our study are defined as: (1) Neiguan (PC6) on Pericardium Meridian of Hand-jueyin group; (2) non-acupoint group.

#### Acupuncture groups

All acupuncture points will be punctured by disposable filiform needles (ANDY Sterile acupuncture needle, 0.25 × 25 mm). After being needled, the points will be punctured again using auxiliary needles 2 mm lateral to the first needle, and to a depth of 2 mm without manual stimulation. HANS-200E electro-acupuncture apparatus (HANS, 2-100Hz, made in Jiangsu, China) is used for electric stimulation at every acupuncture point. Each acupuncture needle and auxiliary needle at each point is connected to a stimulus isolation unit with parameters of 2/15Hz and stimulated for a total of 30 minutes. The stimulation intensity varies from 0.1 mA to 1.0 mA until the patients feel comfortable. De qi sensation [28] will be achieved in the acupuncture groups, through lifting and thrusting combined with twirling and rotating the needles. After retaining the needles for 30 min, all needles are taken out with clean cotton balls to avoid bleeding. Every patient will receive treatments three times a week for a total of 12 treatments, with a 1-day interval every 2 days of treatment.

### Control group

To compare to the acupuncture groups, we randomly selected 20 cases of patients with stable angina pectoris as a control group. They will receive basic treatment including health education and basic drug therapy, but no acupuncture treatment in the first 4 weeks. They will then be provided 12 free sessions of acupuncture treatment after 16 weeks.

### Sample preparation and analyses

#### Neutrophils were selected from peripheral blood

**All** neutrophils are negative selected by the EasySep™ Human Neutrophil Enrichment Kit (Stemcell) from a polymorphonuclear cell-rich fraction of peripheral blood. Unwanted cells are targeted for removal with Tetrameric Antibody Complexes recognizing CD2, CD3, CD9, CD19, CD36, CD56, glycophorin A, and dextran-coated magnetic particles. Labeled cells are separated using an EasySep™ magnet (Stemcell) without the use of columns. Desired cells are then poured off into a new tube.

### RNA-Seq

Total RNAs are extracted by using Trizol reagent (Invitrogen) from 1 × 10^7^ neutrophils selected from 10 ml peripheral blood. RNA libraries are prepared according to the TruSeq RNA Sample Preparation v2 protocol, and the DNA libraries are applied to the cluster generation and sequencing using c-BOT Multiplex re-hybridization plate and Truseq Sbs kit V3. Sequencing will be performed using Illumina Hiseq 2000 (Illumina, USA).

### ChIP-Seq

ChIP is performed by following a protocol from Myers’ laboratory (http://hudsonalpha.org/myers-lab/protocols) with modifications. The neutrophils are crosslinked with 1–2% formaldehyde for 10 min at room temperature. Crosslinking reaction is then stopped by adding 125 mM glycine. The cells are washed with cold PBS twice. 1 × 10^7^ of these cells are collected in 10 ml Farnham lysis buffer (5 mM PIPES pH 8.0/85 mM KCl/0.5% NP-40, supplemented with protease inhibitors) and centrifuged at 4,000 g for 5 min at 4°C. The cell pellet is then washed with 10 ml Farnham lysis buffer, followed by centrifugation. Resulting nuclear pellet is resuspended in 1 ml TE buffer (10 mM Tris-Cl pH 7.7/1 mM EDTA, supplemented with protease inhibitors) and sonicated for 30 min (15 s on/off cycle). Lysates are supplemented with detergents to make 1X RIPA buffer (10 mM Tris-Cl pH 7.7/1 mM EDTA/0.1% SDS/0.1% Na-DOC/1% triton X-100) and centrifuged to remove debris. For each ChIP, 6–8 μg antibodies (H3K4me1, H3K4me2, and H3k27ac) are prebound to 50 μl Dynabeads Protein A (100.02D; Life Technologies) overnight at 4°C. On the next day, the antibody-beads complex is added to chromatin from 2 × 10^7^ cells and further incubated overnight at 4°C. The beads are then washed twice with RIPA buffer, twice with RIPA + 0.3 M NaCl, twice with LiCl buffer (50 mM Tris-Cl pH 7.5/250 mM LiCl/0.5% NP-40/0.5% Na-DOC), and twice with PBS. DNA is eluted and reverse crosslinked in 200 μl elution buffer (1% SDS/0.1 M NaHCO3, supplemented with 20 μg proteinase K) overnight at 65°C. The DNA is purified by QIAquick PCR Purification Kit (QIAGEN) and quantified. DNA and libraries are constructed as described (Wei et al. [29]). All ChIP-Seq samples are sequenced on Illumina HiSeq 2000.

### Measurement of Results

#### Clinical evaluation

The primary measure of effect in our clinical patient is the frequency of angina during the 4 weeks following randomization, which will be measured at the end of every 4 weeks (on weeks 4, 8, 12 and 16). In addition, we will document the following seven parameters: visual analogue scale (VAS), dosage of nitroglycerin tablets, 6-minute walk test (6-MWT), dynamic ECG observation of the changes in ST-T, Seattle angina questionnaire (SAQ), the incidence of cardiovascular episode during the 4 months, self-rating anxiety scale (SAS), and self-rating depression scale (SDS) (Table 1). VAS, dosage of nitroglycerin tablets, and SAQ will be measured one week prior to randomization and at 4, 8, 12 and 16 weeks after randomization. Dynamic ECG observation of the changes in ST-T will be measured before randomization and 4 weeks after randomization. The incidence of cardiovascular episodes during the 4 months will be measured at the end of the 16 weeks in patients who have received the 12 sessions of electro-acupuncture treatment.

**Table 1:** **Trial process chart**

	**Baseline**	**Treatment phase**		**Follow-up phase**		
	**−1 week**	**0 day**	**4 weeks**	**8 weeks**	**12 weeks**	**16 weeks**
**Patients**						
Inclusion and exclusion	*					
Allocate informed consent	*					
Sign the informed consent		*				
Medical history	*					
Laboratory test	*					
Randomization		*				
**Intervention**						
PC6 group (n = 20)		12 sessions of acupuncture				
Non-acupoint point group (n = 20)						
**Comparison**						
Control group (n = 20)						
**Outcomes**						
Angina attacks in four weeks			*	*	*	*
VAS		*	*	*	*	*
Dosage of nitroglycerin		*	*	*	*	*
6-MWT		*	*			
SAQ		*	*	*		
SAS		*	*	*		
SDS		*	*	*		
The incidence of cardiovascular episode during the 4 months						*
Dynamic ECG Observation of the changes of ST-T		*	*			
**Clinic evaluation**						
Safety of electro-acupuncture			*			*
Adverse events			*	*	*	*
Patient’s compliance			*			
Reasons of drop-out withdrawals			*			
**Laboratory evaluation**						
Blood collection		*	*			*
RNA & DNA extraction		*	*			*
RNA-Seq & ChIP-Seq data analysis			*			*

All patients will be asked to record angina diaries one week before randomization (baseline phase) and 4, 8 and 16 weeks after randomization. All participants will receive routine blood, urine, and stool tests, an electrocardiogram (ECG), 24-hour dynamic electrocardiography (DCG), echocardiographic tissue Doppler imaging (TDI), liver function tests (ALT, AST), and kidney function tests (BUN, Scr) before randomization, in order to exclude patients who have serious heart, liver, kidney, or other severe diseases. Routine tests will be run twice before randomization and after the completion of electro-acupuncture treatment.

To guarantee the quality of the study, all acupuncturists are required to attend special training classes. The purpose of these training classes is for acupuncturists to understand all the details of this study. The special training classes focus on theoretical and practical lessons. They will be trained to use the central randomization method, to fill in the case report form, to find the correct points, to manipulate the needles, and to choose the correct electro-acupuncture apparatus. Acupuncturists are qualified to participate in this study only upon completion of all training classes, along with passing the assigned training examination. Additionally, to ensure the quality of this trial, a clinical proctor will check the processes of the trial and document the details at the hospital each week. In the case of any patient dropout, the proctor will further survey the reasons for the patient’s leaving, as well as record the last treatment for that patient as soon as possible.

### Laboratory evaluation

#### Computational analysis for RNA-Seq data

After sequencing with HiSeq2000 (Illumina), raw fastq files are extracted from Illumina BCL using the Illumina CASAVA program. The single-end reads of biological triplicates obtained from each sample are to the rat reference genome (UCSC rn4 assembly) using the TopHat program [30,31]. The Cufflinks program will be used to assemble individual transcripts from RNA-Seq reads that have been aligned to the genome and to qualify the expression level of each transcript. Differential transcripts expression analysis is performed with the Cuffdiff program. The gene’s functional annotation and pathway are analyzed using the DAVID Bioinformatics Resources [32].

#### Computational analysis for ChIP-Seq data

The sequence reads are of 50 bp in length and aligned to reference genome assembly NCBI. The output of the Illumina Analysis Pipeline is then converted to browser extensible data (BED) files detailing the genomic coordinates of each mapped read. To visualize the data on the UCSC genome browser (Karolchik et al. [33]), reads are collected and converted to wiggle (WIG) format using an in-house script.

### Follow-up

Follow-up tests will be conducted 8, 12 and 16 weeks after randomization. The follow-up assessment is designed to evaluate the long-term effects of stable angina pectoris.

### Adverse events

Any adverse events, such as bleeding, hematoma, fainting, serious pain, and local infection, during treatment will be recorded during treatment and in the follow-up period by the acupuncturist. In case of abnormal reactions, the date of appearance and disappearance, the degree of reaction, parameters selected for electro-acupuncture treatment, names of OTC (over the counter) drugs taken outside of the treatment, whether the abnormal reactions were treated, and any other related information will be recorded in detail. Serious adverse events should be reported to the principal investigator immediately. Any medical conditions or diseases present prior to the start of the treatment will be considered abnormal reactions only if they worsen after the start of the treatment. Abnormal test values or results will be considered abnormal reactions only if they cause clinical symptoms, are considered clinically significant, or require treatment.

### Clinic statistical analysis

The results of the intention-to-treat (ITT) analysis will be used to assess the validity of the study as a whole. The per-protocol (PP) analysis results will be used as a reference. The ITT analysis will be used as the main safety assessment technique.

Continuous data will be represented by the average, standard deviation, minimum value, and maximum value, whereas categorical data will be represented by a frequency table. For comparison of the results among the groups, the analysis of variance (ANOVA) test will be used when the data are normally distributed; the Kruskal-Wallis test will be used otherwise. In addition, a chi-square test will be performed for categorical data.

After 4 weeks, different outcomes will be summarized for each group using descriptive statistics, including the median, average, standard deviation, and interquartile range. The differences in angina attacks following the treatment for each group will be analyzed using a paired t-test or a Wilcoxon signed rank test, and a 95% confidence interval will be presented. To assess the difference in the tendency for each visit, a repeated analysis of variance will be performed. A significance level of 5% will be used in all analyses.

VAS, dosage of nitroglycerin tablets, 6-MWT, dynamic ECG observation of the changes of ST-T, SAQ, the incidence of cardiovascular episode during the 4 months, self-rating anxiety scale (SAS), and self-rating depression scale (SDS) will be analyzed using the same techniques used to analyze the validity of the assessment variable. To determine whether there are differences in the distribution of symptomatic changes among the groups, analysis will be performed on each item using the chi-squared test.

All adverse reactions manifested will be listed with detailed explanations. The frequency of abnormal reactions that are correlated with the treatment and abnormal reactions that do not have such correlations will be recorded. A Fisher’s exact test will be performed to determine whether there are any differences among the groups with respect to the incidence of abnormal reactions as reported by the subjects. Furthermore, technical analysis will be performed to identify differences in the degree of severity and in the type of abnormal reactions among the groups.

There is one acupuncturist at each site to do all interventions, which can potentially introduce a clustering effect in this trial. Therefore, we have calculated the intracluster correlation coefficient from the results of this trial and have reported the coefficient [34].

## Discussion

Angina pectoris predisposes a patient to other cardiovascular complications, and affects the patient’s health and quality of life. This study is expected to provide convincing evidence that electro-acupuncture at Neiguan has protective effects against angina, and that many functional genes and pathways contribute to such effects. Moreover, histone modifications are expected to mediate the changes of gene expressions caused by electro-acupuncture treatment.

### Grouping and acupoint selection

There are three acupuncture groups in this trial, including a Neiguan (PC6) group, a non-acupoint group, and a control group. Some reports state that acupuncture on Neiguan may increase the usage of nitroglycerin, just as we have mentioned previously [14]. Therefore, to ensure the quality of research, we asked all participants to use nitroglycerin. In order to avoid interference caused by nitroglycerin’s therapeutic effect, we set up this control group. Our pilot experiment has shown that neither the clinical efficacy nor the molecular changes resulted from acupuncture treatment at Neiguan has been affected by the basic use of drugs. Compared with the control group, we can not only determine the effectiveness of electro-acupuncture on the patients with stable angina pectoris, but also compare the profiles of gene expression and histone modifications. In order to meet the requirements of ethics, the patients in control group will be provided 12 free sessions of electro-acupuncture treatment after 16 weeks. Therefore, we are also able to detect the differences in histone modifications before and after electro-acupuncture treatment at Neiguan for these patients.

The functions of non-acupoint have been reported as controversial by some studies that have shown no difference between acupoint and non-acupoint stimulation [35-37]. To clarify the specificity of acupoint PC6, we set up the non-acupoint group to be a reference point that does not belong to any meridian. We have strategically chosen a non-acupoint as a reference point based on two principles. The first is based on Traditional Chinese Medicine theories and literatures [24]. In most Chinese literature, the preferred non-acupoints is located closely beside therapeutic acupoints and half way between two lines or acupoints [38,39]. The second is based on our previous studies using animal models (unpublished). We have chosen a standard position that does not result in the same effects as the acupoint PC6 when stimulated and has no documented adverse events.

### Target tissue/cell for laboratory study

By using high-throughput sequencing, we can investigate genome-wide gene expressions and histone modifications before and after electro-acupuncture treatment, and then analyze the correlations between acupuncture effects and histone modifications. As a clinical trial, it is not possible to study cardiac tissues of the patients, thus, blood samples are our specimen of choice, and neutrophils are the target cells. Neutrophils, as a hallmark of acute inflammation, has been proven, via autopsy specimens of culprit lesions from acute MI patients, to be vital to acute plaque rupture, demonstrating higher concentrations of activated neutrophils than in those without acute coronary syndrome (ACS) [40]. More recently, the neutrophil/lymphocyte ratio (NLR) has been also proposed as a useful biomarker for predicting cardiovascular risk [41]. Therefore, we have chosen neutrophil as target cells in this study. As a possible result, many pathways found to be effectively activated or repressed by electro-acupuncture treatment might be related to immune response or inflammation.

### Epigenetic marks

Our previous study has demonstrated that electro-acupuncture can promote angiogenesis after myocardial ischemia through H3K9 acetylation regulation at VEGF gene in rat heart tissue [17]. We now focus on two epigenetic marks, H3K4me1/2 and H3K27ac and analyze their genome-wide regulation profiles in this study. Their increased binding to genes in response to acupuncture intervention could indicate an activated chromatin remodeling, and hence a positive regulation for functional gene expressions. Because of budget constraints, we were unable to analyze other epigenetic marks reported to be important for gene regulation, such as H3K4me3, H3K27me3 and H3K9ac.

In conclusion, the results of this study are expected to confirm the effectiveness of electro-acupuncture in the management of stable angina pectoris, and to determine the key role that histone modifications play in gene regulation under acupuncture in stable angina. Our study will provide the first set of gene expression profile for stable angina pectoris and their changes induced by electro-acupuncture. Through in-depth study of histone modifications, we may uncover some molecular evidences of the effects of acupuncture, which can prove useful for basic and clinical researchers, as well as for clinical practitioners.

The trial is sponsored and financially supported by (973 Program, No. 2012CB518501) the Ministry of Science and Technology of China.

## Trial status

The first participants were included on March 28, 2013, and this article was submitted on July 15, 2014. To date, 56 participants have been recruited.
